# A Highly Scalable Peptide-Based Assay System for Proteomics

**DOI:** 10.1371/journal.pone.0037441

**Published:** 2012-06-12

**Authors:** Igor A. Kozlov, Elliot R. Thomsen, Sarah E. Munchel, Patricia Villegas, Petr Capek, Austin J. Gower, Stephanie J. K. Pond, Eugene Chudin, Mark S. Chee

**Affiliations:** Prognosys Biosciences Inc., La Jolla, California, United States of America; University of Pittsburgh, United States of America

## Abstract

We report a scalable and cost-effective technology for generating and screening high-complexity customizable peptide sets. The peptides are made as peptide-cDNA fusions by *in vitro* transcription/translation from pools of DNA templates generated by microarray-based synthesis. This approach enables large custom sets of peptides to be designed *in silico*, manufactured cost-effectively in parallel, and assayed efficiently in a multiplexed fashion. The utility of our peptide-cDNA fusion pools was demonstrated in two activity-based assays designed to discover protease and kinase substrates. In the protease assay, cleaved peptide substrates were separated from uncleaved and identified by digital sequencing of their cognate cDNAs. We screened the 3,011 amino acid HCV proteome for susceptibility to cleavage by the HCV NS3/4A protease and identified all 3 known *trans* cleavage sites with high specificity. In the kinase assay, peptide substrates phosphorylated by tyrosine kinases were captured and identified by sequencing of their cDNAs. We screened a pool of 3,243 peptides against Abl kinase and showed that phosphorylation events detected were specific and consistent with the known substrate preferences of Abl kinase. Our approach is scalable and adaptable to other protein-based assays.

## Introduction

The pace of improvement in DNA sequencing technologies is far outpacing Moore’s Law [1]. As a result, many new whole genome sequences are rapidly becoming available for analysis. This has created a pressing need for new technologies that enable the translation of genomic sequence information into information about protein function at the level of the proteome. Here we address this need by reporting a new approach for producing and assaying large, customizable sets of peptide-cDNA conjugates that combines efficient enzymatic synthesis of *in silico* designed peptide sequences with intrinsically parallel assay formats designed for readout by DNA sequencing.

A variety of molecular biology techniques for the production and screening of large numbers of proteins or peptides linked to their encoding nucleic acid sequences has been developed, primarily to enable the discovery of high affinity binders to targets of interest. These methods, which include phage display [2]–[7], PROfusion™ technology [8], [9], and others [10]–[18], provide means to identify enriched proteins via a nucleic acid tag and avoid the limitations of chemical synthesis (e.g. racemization, limited peptide length, complex multistep synthesis, and use of toxic chemicals) [19].

The sequence diversity of such biologically and biochemically produced constructs is typically generated combinatorially by randomization of nucleic acid sequences [20]–[23]. Although combinatorial approaches are very efficient and straightforward to implement, they are not suitable for representing custom sets of protein sequences of interest, such as the human proteome and its common variations, in a compact form. For example, a random combinatorial library of 10-mer peptides would comprise 20^∧^10, or approximately 10^∧^13, sequences. This is larger than the coding content of the ∼3×10^∧^9 base pair human genome by several orders of magnitude. We address this limitation by making use of microarray-based design and synthesis of custom DNA oligonucleotide sequences to encode specific peptide sequences of interest. The peptide libraries made in this way are smaller than typical random combinatorial libraries, but orders of magnitude larger than conventional custom libraries. Importantly, they can be designed to represent collections of biologically relevant sequences of high interest and utility, such as the human proteome.

Our approach to utilizing peptide-cDNA conjugates also differs from traditional selection-based assays, which use multiple rounds of selection to enrich for a small number of ‘hits’ with desired properties, typically those that bind with the highest affinity [2], [12], [21], [24]. Instead, we seek to obtain information from large numbers of peptides simultaneously in one experiment, generating comprehensive datasets that provide more systematic insight by reporting on a broad spectrum of interactions. In order to illustrate this approach, we designed and developed two types of multiplexed activity assays that employ large custom collections of peptide-cDNA conjugates; one for proteases and one for kinases. Both classes of enzymes have been implicated in a multitude of critical physiological and pathological processes. Therefore, novel multiplexed assays with the capacity to analyze the activity of these enzymes on a proteome-wide scale may find broad application.

Proteases and kinases are involved in two essential regulatory processes, proteolysis and phosphorylation. Proteases activate or terminate biological signaling events through the destruction of proteins [25]–[27]. Similarly, kinases activate or deactivate enzymes, and enable cell signaling, through reversible phosphorylation [28], [29]. Both enzyme classes are involved, often together, in regulation of crucial cellular processes such as DNA replication, cell cycle progression, differentiation, and apoptosis. Misregulation of proteolytic or/and phosphorylation activity is therefore involved in many pathologies including cancer, autoimmune diseases, degenerative diseases, cardiovascular diseases and infectious diseases [30]–[34]. The discovery of potential protease and kinase targets genome-wide may help to elucidate their role in the cell, guide structure and function studies of these enzymes, identify novel drug targets within an important class of ‘druggable’ proteins, and enable the development of new diagnostic tools [28], [29], [31], [35]–[43].

In addition to protease and kinase substrate screening, the approach presented in this report can be used in the selection of peptide substrates for other classes of protein modifying enzymes [44], [45], epitope mapping [46],[47], engineering of new classes of enzymes [48], development of new materials in biotechnology and biomedicine [49], discovery of peptide ligands for proteins [50], semiconductor surfaces [51], [52], specific cell types including cancer cells [4], and identifying agents for efficient and specific delivery of imaging labels [53].

## Results

### Generation of cDNA-peptide Fusions and Assay Optimization

**Figure 1:**
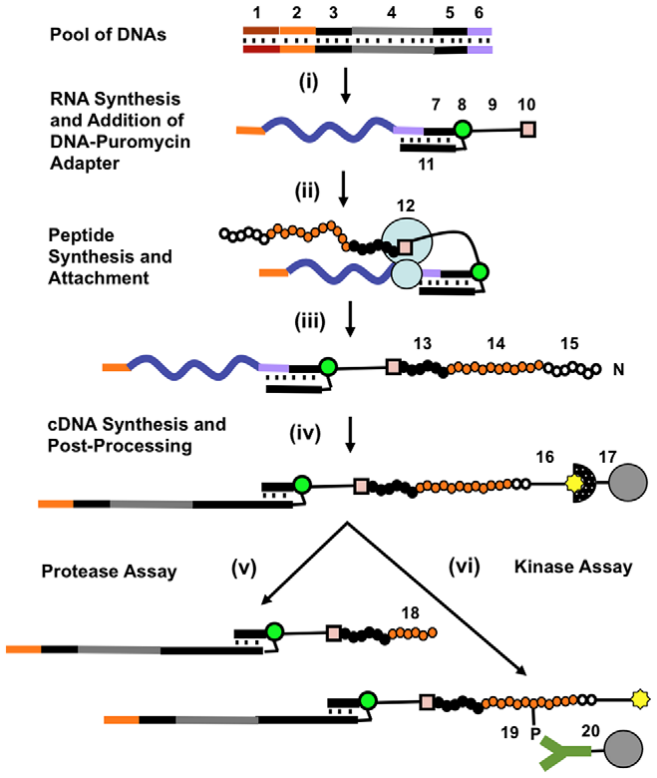
Process for protease and kinase peptide substrate generation and screening. i) DNA templates containing a T7 promoter (**1**), ribosomal binding site (**2**) [100]–[103], sequences coding for N- and C-terminal peptide tags (**3, 5**), a variable region (**4**) coding for custom peptide sequences, and an adapter ligation region (**6**) are transcribed and a DNA adapter is attached to the 3’-end of all RNAs via template-directed ligation [8]. The adapter consists of DNAs (**7**) and (**11**) cross-linked via a psoralen residue (**8**), a linker (**9**), and a 3’-puromycin residue (**10**). **ii–iii**) During in vitro translation, a stalled ribosome (**12**) allows the puromycin residue to enter the ribosome A-site and attach to the C-terminus of the peptide, creating an peptide-RNA fusion [9]. Each peptide in the fusion pool has a custom peptide region (**14**) and two tags (**13**) and (**15**). The C-terminal tag can be used to purify correctly translated full-length peptides. **iv**) The N-terminal peptide sequence is cleaved with TEV protease to expose N-terminal cysteine which is then biotinylated (**16**). The RNA is converted to cDNA by reverse transcription followed by RNAse H treatment [8], and the resulting peptide-cDNA fusions are immobilized on streptavidin coated magnetic beads (**17**). **v**) Protease Assay. In a subsequent assay procedure, the immobilized pool is treated with a protease of interest. The cDNAs attached to cleaved peptides (**18**) are released, collected, amplified, and sequenced. **vi**) Kinase Assay. The pool of peptide-cDNA fusions is released from streptavidin coated magnetic beads (**17**) and treated with a solution containing tyrosine kinase. Phosphorylated peptides (**19**) are immobilized on anti-phosphorotyrosine antibody coated magnetic beads (**20**), specifically eluted with phenyl phosphate, collected, amplified, and sequenced.

Our processes for making and assaying peptide-cDNA pools are illustrated in Fig. 1. The production process that we used is similar to one described earlier for combinatorial peptide libraries [8]. DNA oligonucleotides obtained by microarray-based synthesis were converted into dsDNA templates encoding peptide sequences. The library was then transcribed to produce mRNAs, and a DNA adapter conjugated to puromycin was ligated to the 3’ end of the mRNAs. During subsequent *in vitro* translation of mRNAs, puromycin entered the ribosome A-site and attached to the C-terminus of the nascent peptide, forming a covalent fusion between the peptide and its encoding mRNA. Peptides were then cleaved at a specific location by TEV protease to generate an N-terminal cysteine, which was biotinylated to allow capture and purification of the fusion molecules. The mRNA portion of the fusion was then converted into cDNA by reverse transcription and RNAse H treatment. Alternatively, conversion to cDNA can be done after *in vitro* translation and prior to TEV protease cleavage.

**Figure 2:**
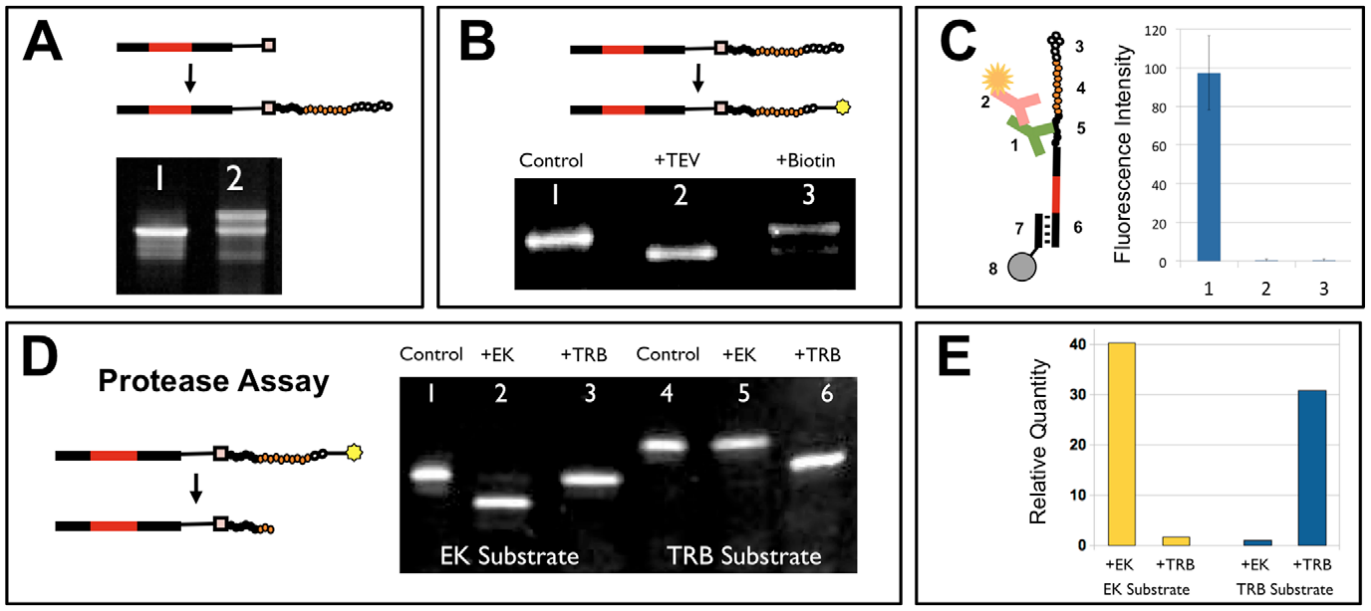
Individual steps of the peptide substrate generation and screening process. A) Peptides were attached to RNA as shown in Fig. 1, steps (**ii–iii**) and reaction products analyzed by denaturing polyacrylamide gel electrophoresis (PAGE, Novex 15% TBE-Urea gels stained with SYBR Gold, Life Technologies). PAGE Lane 1: starting RNA with puromycin adapter; Lane 2: product of peptide attachment. **B**) Peptides were biotinylated at their N-terminus as shown in Fig. 1, step (**iv**). PAGE Lane 1: starting nucleic acid-peptide fusion; Lane 2: product of cleavage with TEV protease; Lane 3: product of reaction of biotin-PEG attachment to the N-terminal cysteine. **C**) Presence of fully translated peptide (**3–4–5**) was confirmed by detection of the C-terminal FLAG tag (**5**) by mouse anti-FLAG antibodies (Sigma) (**1**) and Cy3 labeled goat anti-mouse antibodies (Jackson ImmunoResearch) (**2**). Peptide-RNA fusions were captured by hybridization of a common region (**6**) to oligonucleotides (**7**) attached to beads (**8**). Beads were imaged using a DM6000B automated fluorescence microscope and imaging system (Leica). Bar 1 represents *in vitro* translation in the presence of puromycin modified template; Bar 2 is a no template control; Bar 3 is puromycin modified template without *in vitro* translation. **D**) Protease assay results. The process is shown in Fig. 1, step (**v**). Individual peptide-RNA fusions containing peptide substrates for enterokinase (EK) and thrombin (TRB) proteases (AGDDDDKAG and GLVPRGSAG respectively) were cleaved by individual proteases and the reaction products were analyzed by PAGE. Lanes 1–3 show results for peptide substrate for enterokinase (EK), lanes 4–6 show results for peptide substrate for thrombin (TRB). **E**) Quantitative RT-PCR analysis of the assays shown in panel D, lanes 2–3 and 5–6. The Y-axis represents relative DNA quantities calculated from qPCR C_t_ values.

We first established all the steps of peptide-cDNA fusion formation using individually synthesized DNA templates (Fig. 2A–B). A critical step of the process is peptide-RNA fusion formation during *in vitro* translation. The conversion efficiency of RNA-puromycin molecules to RNA-peptide fusions was studied and estimated by gel-electrophoresis to be about 50% (Fig. 2A). Cleavage of the peptide-RNA fusion by TEV protease and subsequent biotinylation was monitored in a similar way (Fig. 2B). In order to confirm that full-length peptides were synthesized, the RNA-peptide molecules were captured on magnetic beads and the C-terminal tag detected (Fig. 2C). In a single-substrate protease assay (Fig. 1, step (v)), peptide-RNA fusions coding for enterokinase and thrombin substrates were captured separately on streptavidin coated beads and subjected to cleavage with individual proteases. Cleavage was confirmed by a change in gel electrophoretic mobility (Fig. 2D) and RNA quantification (Fig. 2E). Similarly, in a single-substrate kinase assay (Fig. 1, step (vi)), a single cDNA-peptide fusion containing Abl substrate (GEAIYAAPFA) was treated with Abl kinase. Phosphorylated cDNA-peptide fusion was then captured on magnetic beads coated with anti-phosphotyrosine antibodies [40], [54] and subsequently eluted and quantitated by qPCR. We detected over 128-fold enrichment of the phosphorylated peptide over non-phosphorylated negative control.

### Multiplexed Protease Assay

In order to validate the peptide-cDNA conjugate pools in a multiplexed functional assay, and demonstrate the scalability of our technology, we studied the substrate specificity of NS3/4A (hepacivirin) protease of hepatitis C virus (HCV), a major cause of morbidity and mortality worldwide [55]. The viral genome is translated into a single polyprotein of 3,011 amino acids that is cleaved at four sites by the HCV NS3/4A protease [56]. This protease is essential for viral replication and the formation of infectious viral particles, and thus has been considered an attractive target for HCV therapy [57]–[61]. Nevertheless, there is relatively little data available regarding the substrate specificity of the HCV NS3/4A protease. Only the four biologically relevant peptide substrates have been reported [62] and listed in the MEROPS protease database as 8-mer peptides [26]: NS3/4A-NS4A (^1654^EVVT↓STWV^1661^), NS4A-NS4B (^1708^MEEC↓SQHL^1715^), NS4B-NS5A (^1969^TTPC↓SGSW^1976^) and NS5A-NS5B (^2417^VVCC↓SMSY^2424^).

**Figure 3:**
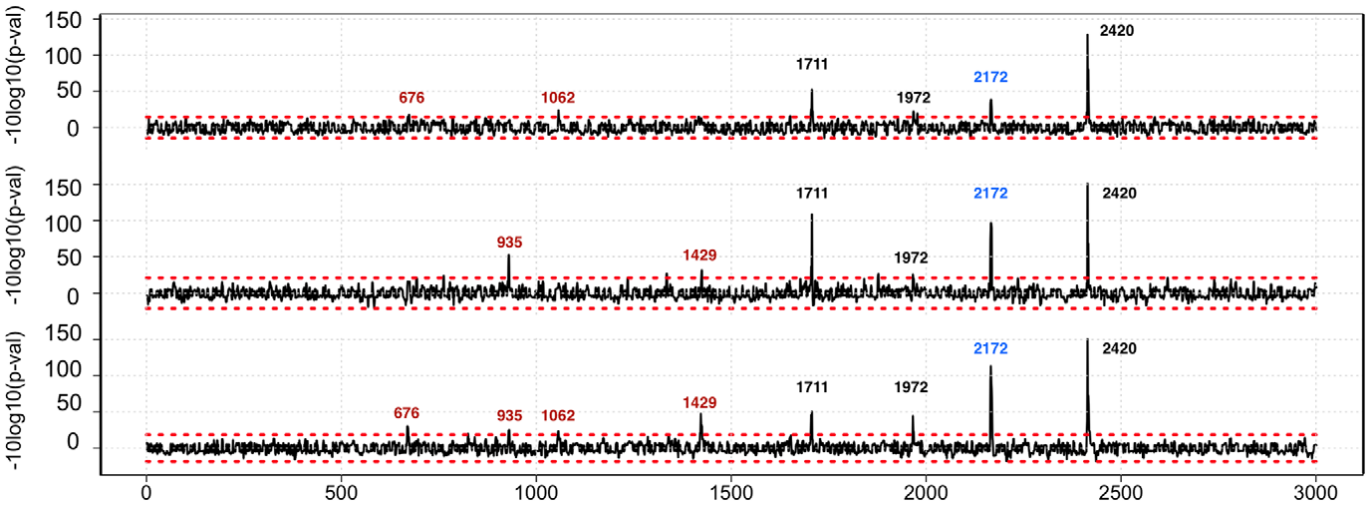
HCV NS3/4A protease cleavage map of the 3,011 amino acid sequence of HCV polyprotein. Cleavage sites were identified by assaying 3,001 overlapping 10-mer peptides covering the entire 3,011 amino acid sequence of HCV polyprotein. The graphs represent a response to HCV NS3/4A protease treatment for three experiments (see Methods). The X-axis shows coordinates along the HCV polyprotein. The Y–axis represents log-transformed p-values with sign showing directionality. Z-scores were transformed using -sign(z)x10xLog10(Pz) where Pz is p-value of derived from standard normal distribution. Red dotted lines mark cutoffs computed from the maximum transformed Z-score in the negative direction. Published HCV NS3/4A protease cleavage sites (1711, 1972, and 2420) are numbered in black. A new site (2172) is labeled in blue. The numbers in the labels represent the position of the P1 amino acid (amino acid position at the C-terminus of the cleaved peptide bond) in the HCV polyprotein. Assay signals that did not reach significance in all three experimental conditions (15, 30, and 60 minutes protease treatments from the top to the bottom panels respectively), but were detected in at least two experimental conditions are labeled in red. The numbers in these labels represent the amino acid position in the HCV polyprotein corresponding to the maximum of the peaks.

**Figure 4:**
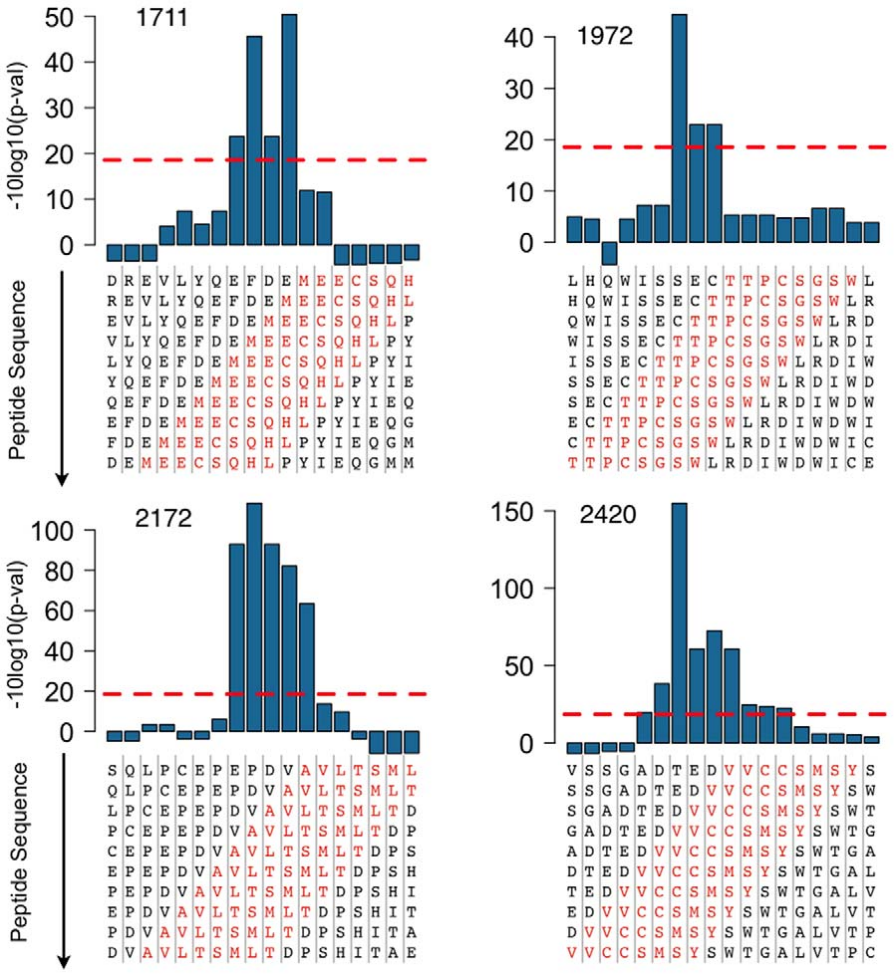
Results from peptides spanning the cleavage sites 1711, 1972, 2172, and 2420. The plots show data from sets of overlapping 10-mer peptides representing portions of the HCV polyprotein where protease activity was detected at all three time points in our assay (Fig. 3). Red dotted lines mark cutoffs computed from the maximum transformed Z-score in the negative direction. The Y–axis represents log-transformed p-values with the sign showing directionality. Z-scores were transformed using -sign(z)x10xLog10(Pz) where Pz is the p-value derived from standard normal distribution. Because the peptide sequences are shifted in increments of 1 amino acid, several adjacent peptides contain sufficient recognition sequences to be cleaved. Peptide sequences are written vertically and the HCV NS3/4A protease recognition sequences corresponding to identified P4-P4’ positions are shown in red.

We produced a set of 3,001 DNA templates coding for 10-mer overlapping peptide sequences derived from the HCV polyprotein with a step of one amino acid. Next, we converted these templates into corresponding peptide-cDNA conjugates and confirmed that all the expected conjugates were present, by sequencing following purification via N-terminal biotin capture (see Methods for details). The pool of cDNA-peptide fusions was immobilized on magnetic beads and used to assay protease activity (see Methods). The HCV NS3/4A protease cleavage data for the 3,001-plex peptide set are presented in Fig. 3. Although the overall background was low, the presence of a few sporadic peaks indicated that there is some random noise in this assay. Therefore, we used a more stringent criterion of requiring that a signal be present at all three time points in order to be considered a clear positive. By this criterion, we detected cleavage of the three known *trans* sites published in the MEROPS database (^1708^MEEC↓SQHL^1715^, ^1969^TTPC↓SGSW^1976^, and ^2417^VVCC↓SMSY^2424^, labeled 1711, 1972, and 2420 respectively in Fig. 3). The fourth NS3/4A-NS4A site (^1654^EVVT↓STWV^1661^) was not detected in our assay. This site is cleaved in *cis*, i.e. via an intramolecular cleavage event, and therefore would not be expected to be detected [63], [64]. We also identified a cleavage site (^2168^VAVLT↓SMLTD^2177^ ) that was not reported previously (labeled as 2172 in Fig. 3). Results from individual peptides that span the four cleavage sites are shown in Fig. 4. In order to verify our results, and to evaluate the significance of the sporadically detected sites (labeled in red in Fig. 3), individual peptides were chemically made, treated with HCV NS3/4A protease, and analyzed by HPLC and LCMS (See Text S1 and Table S1 for details). All four positives were confirmed, and one of two sporadic positives tested was detected as cleaved. However, only the three known positives were detected by HPLC, which is less sensitive than LCMS, indicating that the newly detected sites are cleaved less efficiently. A more detailed analysis of NS3/4A specificity was carried out using a collection of 8-mer peptides and is reported in an accompanying paper [65].

### Multiplexed Protein Kinase Assay

The ability to discover potential protein kinase substrates and assay them at high resolution across the proteome would help guide experiments to elucidate the functional roles of specific kinases [37]–[43]. In order to demonstrate the feasibility of such an approach, we designed and synthesized a 3,243-plex set of 10-mer peptides for kinase assays. The identity of peptide-cDNA conjugates was confirmed by sequencing as described for the protease assay above. The pool included kinase peptide substrates and negative controls published earlier [66]–[70], and kinase peptide substrates obtained from phosphoroproteomics databases [70]–[73]. Additionally, we included systematic variations of the consensus sequence for Abl (GEAIYAAPFA) [74], [75], varying all amino acids in each sequence position, one at a time. Finally, the pool included the entire sequences of cortactin, Flt3, and Src, proteins that each contain several known phosphorylation sites [70], represented by overlapping 10-mer peptides with a step of two amino acids.

**Figure 5:**
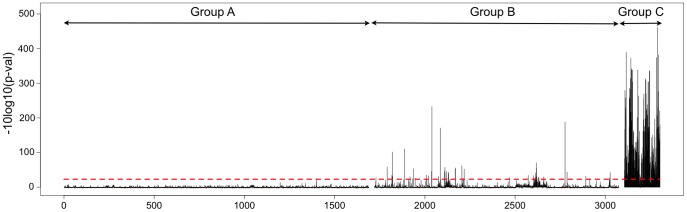
Abl kinase phosphorylation map of a 3,243-plex peptide substrate pool. Each bar along the X-axis corresponds to a signal from an individual peptide in the pool. In order to simplify visualization, peptides were split into three groups. Group A contains 1,690 peptides that do not have any tyrosine residues and includes 19 negative control peptides that are derived from a known Abl kinase peptide substrate (GEAIYAAPFA) where the tyrosine residue was changed to all remaining 19 natural amino acids while the rest of the sequence was kept constant. Group B contains 1,355 peptides that have at least one tyrosine residue. Group C contains 198 peptides that are known to be substrates for Abl kinase or derived from a known Abl kinase peptide substrate (GEAIYAAPFA) where amino acid residues in each individual position (except the tyrosine residue) were systematically changed to all remaining 19 natural amino acids while the rest of the sequence was kept constant. The Y–axis represents log-transformed p-values with sign showing directionality. Red dotted lines mark cutoffs corresponding to the maximum Z-score observed for peptides lacking tyrosine. Z-scores were transformed using -sign(z)x10xLog10(Pz) where Pz is the p-value derived from standard normal distribution.

**Figure 6:**
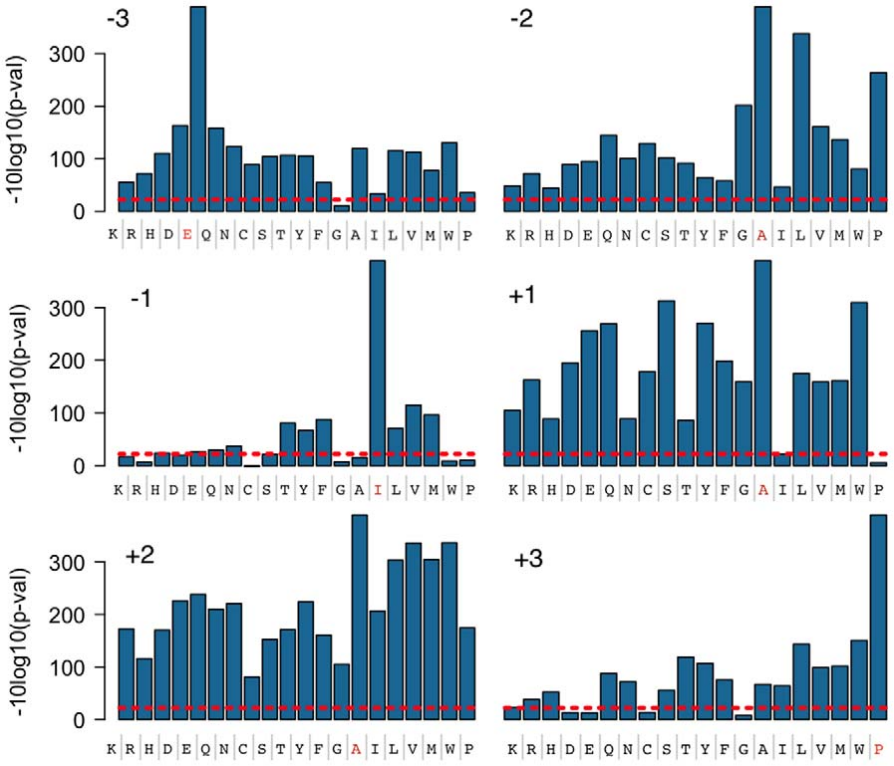
Sensitivity of Abl kinase to sequence variation as a function of position. Each panel shows the effect of varying the amino acid sequence at one position in the Abl kinase substrate GEAIYAAPFA. The position that is varied (top left of each panel) is shown relative to the central tyrosine, which is in the ‘0’ position. The amino acid that is substituted at the position of interest is shown on the X-axis, with the reference sequence shown in red. The Y–axis represents log-transformed p-values with sign showing directionality. The data are a subset of the 3,243-plex pool data shown in Fig. 5 (Group C).

We used Src (p60), Abl, Lck, Flt3, Kit, and Jak2 tyrosine kinases in assays with the 3,243-plex peptide set. Preliminary analysis revealed distinct patterns of phosphorylation for each of the kinases tested (data not shown). In this report we present results for Abl kinase (Fig. 5). Our pool included 1,690 peptides that contained serine and threonine but no tyrosine residues (Group A, Fig. 5), serving as a large collection of negative controls in the experiments that measure only phosphorylation by tyrosine kinases. Fig. 5 shows that the magnitudes of p-values for 1,690 negative peptides (group A) are clearly separated by orders of magnitude from p-values of positive controls (group C), indicating very high specificity and sensitivity of the assay. Group C (Fig. 5) contained 198 peptides that were i) known to be substrates for Abl kinase; or ii) systematic amino acid variants of a known Abl kinase peptide substrate (GEAIYAAPFA). All known Abl kinase substrates and the majority of the mutant peptides derived from the GEAIYAAPFA wild type sequence were phosphorylated by Abl kinase in our assay (a total 90% of Group C). More detailed results for peptides derived from GEAIYAAPFA, in which amino acid residues in the −3, −2, −1 +1, +2, and +3 positions flanking the tyrosine phosphorylation site were systematically varied, are shown in Fig. 6. The sequence preference observed for Abl kinase is consistent with published data and known in vivo substrates [74], [75]. For example, we observed a strong requirement for isoleucine at the −1 position and proline at the +3 position. Group B contained 1,355 peptides that have at least one tyrosine residue and therefore are potential substrates for Abl kinase. We found that 53 peptides from Group B (4%) were phosphorylated by Abl kinase. Of these, 38 peptides were previously reported as substrates for Src, Lck, Flt3, and EphB2 kinases [69]. The 15 remaining phosphorylated peptides included 6 peptides derived from cortactin, Src, and Flt3 proteins: GTEPEPVYSM and GLAYATEAVY (derived from the cortactin protein walk); SEEPIYIVTE and LVQLYAVVSE (derived from the Src protein walk); YATIGVCLLF and LSGPIYLIFE (derived from the Flt3 protein walk); and 9 previously unassigned peptides.

## Discussion

An intrinsically parallel way to convert an in silico specification to a large collection of reagents is a technological cornerstone of modern genomics approaches. For example, in applications such as large-scale genotyping and targeted enrichment of genomic regions, many oligonucleotides, made at low cost using parallel synthesis technologies, are utilized in large sets. However, making and assaying custom sets of thousands to millions of peptides is a major technical barrier to proteome-scale analysis using peptide libraries. Even a set of 1,000 peptides is quite large by the standards of conventional technologies. Although such collections can be made, they are expensive, and they would not necessarily be usable for parallel, low-cost analysis. In this work we adapted and integrated existing genomics technologies to develop a new technology for proteomics. In our approach, large collections of custom peptide sequences, designed *in silico,* are made as peptide-cDNA conjugates and then used in highly multiplexed assays.

Our rationale for making the peptides as conjugates with their encoding DNA sequences was to enable the readout of results by digital nucleic acid sequencing. Together with the development of intrinsically parallel ways to assay pools of peptides, this ensures that our entire process is highly scalable and cost-efficient. Given that not all substrates in a large pool are cleaved, or phosphorylated, and therefore do not generate signal, the capacity of the current generation of massively parallel DNA sequencers is already sufficient to screen peptide sets of ≥1 million at a low cost per data point. As a readout technology, digital sequencing is flexible, with the ability to adjust sensitivity and dynamic range by the number of reads obtained. Our method is also quite specific. It benefits from the single nucleotide discrimination provided by DNA sequencing, which allows us to filter out the small fraction of data points that may have been produced by incorrectly encoded peptides. At the peptide level, the ability to tile through a protein sequence with overlapping peptides provides redundancy, which also improves sensitivity and specificity. Multiple peptides spanning a protein site of interest can each generate a signal, as shown in Figs. 4 and 6, which helps with positive signal identification during data analysis.

We showed that our peptide sets are functional in protease assays by screening the entire HCV polyprotein and identifying a small number of ‘hits’ for subsequent confirmation. Our approach enabled *de novo* identification of all three known *trans*-cleavage sites of the HCV NS3/4A protease in the 3,011 amino acid long HCV polyprotein, recapitulating results from the literature [56]. We also confirmed two new sites (^2168^VAVLT↓SMLTD^2177^ and ^672^QVLPC↓SFTTL^681^). Similarly, we demonstrated the feasibility of discovering kinase substrates by screening the potential phosphoproteome, using peptides designed from genomic sequence information. The Abl kinase assay results, including a consensus sequence mutational analysis (Fig. 6) and Abl reactivity on known substrates of other tyrosine kinases, provided a characterization of substrate preferences. These results show that one possible application of our technology is to identify sets of substrates that can be used as specificity ‘fingerprints’ to maximize the power to distinguish the activity of different kinases or proteases with similar specificities.

The new substrate sequences for HCV NS3/4A protease (^2168^VAVLT↓SMLTD^2177^ and ^672^QVLPC↓SFTTL^681^) identified in our assay are located in the central region of NS5A RNA polymerase and in the E2 protein respectively and may not be readily accessible for proteolysis under physiological conditions in vivo. Therefore, these sites are unlikely to be biologically relevant. However, our results also illustrate the ability to identify *in vivo* relevant and functionally significant sites. Our kinase assay, as well as several in vivo global studies using mass spectrometry, have identified GTEPEPVYSM as a phosphorylation site in cortractin [76], [77]. This phosphorylated tyrosine is one of the key regulatory points of cortactin in response to stress [78]. These two examples from the opposite sides of spectrum illustrate one limitation of our approach. Because we represent a protein sequence in the form of multiple short peptides and carry out assays *in vitro*, there may be significant conformational and accessibility differences that limit the ability to extrapolate results to the protein and *in vivo* context. Therefore, additional filtering may be required to distinguish sites that are functional *in vivo* from those that are active only *in vitro* on the peptide level. For example, one approach may be to make use of 3-dimensional protein structural information to compute theoretical accessibility of individual peptides mapped on the structure.

Another limitation is that our peptides are produced without post-translation modifications, and therefore in some cases may not reflect the situation *in vivo*, where proteins undergo a wide variety of modifications, such as acetylation, phosphorylation, glycosylation, or disulfide bridge formation [79], [80]; although our production protocol could be modified to make peptide-cDNA libraries with modified or unnatural amino acids [81]–[83]. Additionally, a possible source of biases may be interference of cDNA tags with enzymatic activity on peptides leading to false-negatives. However, we have not observed this to be a problem since signals from all positive controls in both assays were detected. Nevertheless, the sheer breadth of sequences that we are now able to assay should make this technology a useful discovery tool. The ability to survey large tracts of sequences, as well as systematic variations that can be designed *in silico*, enables comprehensive analysis of sequence dependences, and can provide insights into function (see accompanying paper [65]).

Despite these limitations, the ability to screen large amounts of sequences rapidly and efficiently enables the discovery of new protease and kinase targets. Importantly, because of the parallel design of our approach, even orders of magnitude increase in the number of peptide–cDNA conjugates that are made and assayed would have a relatively modest impact on cost. Prior to our technology, approaches for the identification of protease and kinase substrates have been developed that employ individually synthesized peptide substrates [84]–[89]. The high cost of synthesizing peptides individually limits these approaches for large-scale screening of sequences. The cost of peptides produced by parallel synthesis is much lower [90]–[92] but the current price of peptide microarrays is still a barrier to large scale studies [93]. Recently, a method for the production of low cost oligonucleotide libraries has been developed in parallel with our approach [94] and used to generate a phage displayed peptide library with 413,000 members [46]. This library was successfully used to identify autoantigens, and also served to illustrate the power of parallel approaches. In principle, phage libraries could also be used for protein activity assays, and our peptide-cDNA conjugate libraries for binding assays. However, peptide-cDNA conjugates are much lower in molecular weight, which reduces the opportunity for non-specific interactions, and may make it more straightforward to adapt to different types of assays. Also, our process can be carried out completely *in vitro*, which may help to avoid cloning and propagation biases that may occur with phage libraries.

The approach presented in this report should scale to sets that are orders of magnitude larger. In order to realize the full potential of our approach, we plan to scale up the level of multiplexing and also develop additional assays, such as assays for serine and threonine kinases. In conclusion, we believe that our technology has the potential to open up new opportunities for designing and using large custom peptide sets. We expect that it will stimulate the development and application of a variety of proteome-wide assays for basic and applied research.

## Methods

### DNA Pool Production

DNA pools were synthesized via a microarray based method that is similar to a previously published approach [46]. Briefly, single-stranded oligonucleotide templates (ssDNAs) encoding the peptide sequences were synthesized on the array surface and phosphorylated using T4 polynucleotide kinase. Each of the templates has a universal 5-mer site at the 5’-end for splint ligation and a universal primer site at the 3’-end coding for a common tag. A universal DNA sequence was ligated to all oligonucleotides on the array using T4 DNA ligase and a splint oligonucleotide. This sequence contains an untranslated region (UTR) with a T7 promoter and a ribosomal binding site (RBS). Next, a universal primer was hybridized to the 3’-end of all sequences on the array. The primer was extended by DNA polymerase and the resulting ssDNAs were eluted from the array and amplified by PCR.

### Synthesis of Puromycin DNA Adaptor

The DNA adaptor consists of a DNA oligonucleotide with a 5’ phosphate modification and a 3’ puromycin residue attached via a polyethylene glycol (PEG) linker [95], and a DNA oligonucleotide with a 5’ psoralen residue, complementary to the puromycin modified oligonucleotide and the 3’ end of RNAs. The two oligonucleotides were annealed and covalently linked by psoralen-induced photo-crosslinking [8], [96].

### Synthesis of RNA-puromycin Molecules

Amplified DNA pools were transcribed using a standard protocol (AmpliScribe T7 Flash, EpiCentre). Transcription reactions were cleaned up using Qiagen RNeasy Mini Kit and quantified. The DNA adaptor was attached to the 3’ end of RNAs via template directed ligation [8]. Ligated RNA molecules were purified using 15% TBE-Urea denaturing gel.

### Generation of cDNA Peptide Fusions

The RNA-puromycin molecules were translated *in vitro*, and then converted into cDNA following published protocols [8]. The peptide-cDNA pools were treated with RNAse H and RNAse A, and then captured via complementary oligonucleotides attached to silica beads. All peptide-cDNA conjugates contained a modified TEV protease cleavage sequence (GENLYFQCA) at the N-terminus. Treatment with AcTEV protease (Invitrogen) exposed an N-terminal cysteine residue [97] that was then modified with biotin-PEG in the form of a thioester [98]. Next, the biotinylated products were eluted from the beads for use in either the protease or kinase assay as described below. A portion of the pool was set aside for sequencing to characterize peptide representation in the starting peptide-cDNA pool. In order to confirm that all peptide-cDNA conjugates were present in the multiplexed pools, the pools were immobilized on streptavidin beads via biotin attached to the peptide portion of the conjugates (Fig. 1, step iv). After washing with PBS with 0.05% Tween 20 to remove unbound conjugates, the immobilized conjugates were eluted with 95% formamide for 10 min at 95°C, PCR amplified, and sequenced.

### Protease Assay

Solution containing peptide-cDNA conjugates (10 µL of ∼0.1 µM in water) was diluted two-fold with Superblock (Thermo Scientific). A magnetic streptavidin bead suspension (2 µL of 1% solids, Seradyn) was added and the mixture was incubated for 15 minutes at room temperature. The beads were washed three times with 100 µL 1× PBS 0.05% Tween 20 to remove unbound conjugates. Next, the beads were suspended in 10 µL of protease solution with HCV NS3/4A protease (Anaspec) at 20 µg/mL in 50 mM Tris HCl pH 7.5 buffer containing 100 mM NaCl, 10 mM DTT, 20% glycerol. The immobilized conjugates were exposed to the solution at 37°C with samples being collected at 15, 30, and 60 minutes. Negative controls were subjected to identical conditions without protease present. Samples were taken from the supernatant, filtered to remove any leftover beads, and prepared for sequencing as described below.

### Kinase Assay

The peptide-cDNA conjugates (∼0.1 µM) were treated with 20 units of Abl kinase (New England Biolabs) in 50 mM Tris-HCl pH 7.5 buffer containing 10 mM MgCl_2_, 2 mM DTT, 0.1 mM EDTA, 0.01% Brij-35, and 750 µM ATP. Untreated sample was used as a negative control and was processed in parallel in the absence of kinase. Reactions were carried out at 32°C for 1 hour. After phosphorylation, peptide-cDNA conjugates were captured on streptavidin beads, followed by elution in water at 95°C. Phosphorylated peptide-cDNA molecules were captured from each sample using magnetic beads with immobilized anti-phosphotyrosine antibodies [40], [54] (Millipore). Enriched phosphorylated peptides were eluted with 1 mM phenyl phosphate solution in PBS with 0.05% Tween 20 and prepared for sequencing as described below.

### Library Preparation and Sequencing

DNA libraries produced by the protease or kinase assay were amplified, and sequencing adapters incorporated, by PCR [99]. Typically, 25 cycles of PCR were needed to produce sufficient amounts of material for sequencing. When appropriate, unique barcodes were incorporated during PCR and multiple libraries were pooled prior to purification. PCR products were then purified by PAGE on 6% polyacrylamide TBE gels (Invitrogen), eluted in 0.1X TE buffer and precipitated with ethanol in the presence of 100 mM sodium acetate and 1 µL of Pellet Paint (EMD Chemicals). The PCR product was dissolved in water and sequenced using a Genome Analyzer IIx following a standard protocol (Illumina).

### Protease Assay Data Analysis

Prior to statistical analysis, products made from DNA templates with spontaneous mutations (arising from errors in DNA synthesis or nucleic acid amplification) were identified by sequencing data analysis and filtered out *in silico.* Three pairs of protease treated and untreated samples (15, 30, and 60 minutes) were compared in each experiment (see Raw Assay Data S1). In order to reduce the impact of noise at low signal levels, raw sequence counts were log2 transformed after adding 256 virtual counts. A smoothing spline was then fitted using the smooth.spline routine from the R statistical package. The residuals from the fit were converted to Z-scores after scaling by a standard deviation estimated via median absolute deviation. Z-scores were further smoothed along the HCV polyprotein sequence by a 3 amino acid moving median filter. Under a no cleavage hypothesis the distribution of resulting Z-scores is expected to be symmetric. Therefore, we used the absolute value of the maximum negative Z-score as a cutoff to call cleavage events in each pair of samples.

### Kinase Assay Data Analysis

Prior to statistical analysis, products made from DNA templates with mutations were filtered out *in silico* as described above for protease assay. Four Abl kinase treated and untreated pairs of samples were compared (see Raw Assay Data S2). Raw sequence counts were log2 transformed after adding 512 virtual counts to reduce the impact of noise. A smoothing spline was fitted with df = 4 argument to the smooth.spline routine and zero weight was given to points corresponding to known Abl substrates. The residuals were converted to Z-scores the same way described above for the protease assay. We used the maximum Z-score of peptides containing no tyrosine as a cutoff for rejecting no phosphorylation hypothesis.

## Supporting Information

Text S1 **Confirmation of Putative NS3/4A Protease Substrates.** Experimental design, methods, and results of cleavage analysis performed on chemically synthesized peptides treated with NS3/4A protease.
(DOCX)

Table S1 **Results for Confirmation of Putative NS3/4A Protease Substrates.** A plus sign (+) indicates that cleavage by HCV NS3/4A protease was observed. A minus sign (−) indicates that cleavage was not observed. N/A indicates a sample that was not analyzed. The column “Cleavage Site” shows positions of the cleavage sites identified by LCMS assay (designated with arrows). The “Peptide Assay” column summarizes results of our assay shown in Fig. 3.
(DOCX)

Raw Assay Data S1 **Protease Assay Raw Data (HCV NS3/4A protease).** Sequencing counts for each peptide and specific treatment condition are presented. Three pairs of protease treated and untreated samples (15, 30, and 60 minutes) were compared in each experiment (15, 30, and 60 HCV plus/minus, respectively).
(CSV)

Raw Assay Data S2 **Kinase Assay Raw Data (Abl kinase).** Sequencing counts for each peptide and specific treatment condition are presented. Four Abl kinase treated and untreated pairs of samples were compared (Abl plus/minus 1–4).
(CSV)
