# Supplementary material for: A Highly Scalable Peptide-Based Assay System for Proteomics
Source: PLoS One. 2012 Jun 12;7(6):e37441. doi: 10.1371/journal.pone.0037441 (PMC3373263; doi:10.1371/journal.pone.0037441)
Supplement: Text S1 — Confirmation of Putative NS3/4A Protease Substrates. Experimental design, methods, and results of cleavage analysis performed on chemically synthesized peptides treated with NS3/4A protease. (DOCX) [file pone.0037441.s001.docx]

**SUPPORTING INFORMATION**

**Confirmation of Putative NS3/4A Protease Substrates**

Individual chemically synthesized peptides were treated with NS3/4A protease and analyzed first by HPLC and then by LC with MS detection (LCMS). Peptides tested included known cleavage sites that are also positive in our assay (3 peptides containing the known NS3/4A *trans* cleavage sites), negatives in our assay (the known NS3/4A *cis* cleavage site as well as 2 peptides that were randomly chosen from peptides with no significant signal), the newly detected 2172 site, and two of the four sporadically detected sites (676 and 1429 in Fig. 3). The sequences of the peptides tested and the assay results are shown in Table S1. Using the HPLC assay, only the peptides corresponding to known HCV substrates were detected as being cleaved. Using LCMS, a more sensitive method, the peptides corresponding to known substrates, the newly identified substrate (2172), and one of the two sporadically detected sites tested (676), were identified as being cleaved. MS analysis allowed identification of the cleavage site for the new substrates (^672^QVLPC↓SFTTL^681^ and ^2168^VAVLT↓SMLTD^2177^), which are in agreement with known NS3/4A protease cleavage preferences (C↓S and T↓S).

**Methods**

Chemically synthesized peptides were obtained from Sigma PEPscreen. Two amino acids (GY) were added to the C-terminus of all peptides to improve UV absorbance detection during HPLC. All peptides were subjected to NS3/4A protease treatment and first analyzed on HPLC with UV detection for potential cleavage products, and then, to increase the sensitivity of detection, all samples were reanalyzed using LCMS (single quadrupole MS). Peptide cleavage with HCV NS3/4A protease was performed as follows: 0.5 μL of HCV NS3/4A (200 μg/mL, Anaspec) was added to a solution of synthetic peptide (0.1mg/mL) in HCV reaction buffer (50 mM Tris HCl pH 7.5, 100 mM NaCl, 10mM DTT, 20% glycerol) with 1% DMSO, and incubated for 3h at 32 °C. An additional 0.5 μL of HCV NS3/4A /4A (200 μg/mL) was added and incubation continued for another 3h at 32 °C. Samples were diluted into water (1:1 to 1:4 dilution) prior to LCMS analysis. Cleaved peptide samples were analyzed on an Agilent 1200 series HPLC and Agilent 1100 series LCMS. Liquid chromatography was performed on a Poroshell 300 2.1x75mm 5μ C18 column with 5% to 45% B over 13 min gradient (A: 0.1% HCOOH in water; B: 0.1% HCOOH in CH3CN) and a 0.2 mL/min flow rate. MS was performed with positive mode detection and m/z 150 – 1600 scan range.
