# Supplementary material for: A Highly Scalable Peptide-Based Assay System for Proteomics
Source: PLoS One. 2012 Jun 12;7(6):e37441. doi: 10.1371/journal.pone.0037441 (PMC3373263; doi:10.1371/journal.pone.0037441)
Supplement: Table S1 — Results for Confirmation of Putative NS3/4A Protease Substrates. A plus sign (+) indicates that cleavage by HCV NS3/4A protease was observed. A minus sign (−) indicates that cleavage was not observed. N/A indicates a sample that was not analyzed. The column “Cleavage Site” shows positions of the cleavage sites identified by LCMS assay (designated with arrows). The “Peptide Assay” column summarizes results of our assay shown in Fig. 3. (DOCX) [file pone.0037441.s002.docx]

**Table S1. Results for Confirmation of Putative NS3/4A Protease Substrates.** A plus sign (+) indicates that cleavage by HCV NS3/4A protease was observed. A minus sign (-) indicates that cleavage was not observed. N/A indicates a sample that was not analyzed. The column “Cleavage Site” shows positions of the cleavage sites identified by LCMS assay (designated with arrows). The “Peptide Assay” column summarizes results of our assay shown in Fig. 3.

| Peptide ID | Sequence | Peptide  Assay | HPLC Assay | LCMS Assay | Cleavage Site | Comments |
| --- | --- | --- | --- | --- | --- | --- |
| 1711 | EMEECSQHLPGY | + | + | + | EMEEC↓SQHLPGY | Known *trans*-substrate |
| 1972 | CTTPCSGSWLGY | + | + | + | CTTPC↓SGSWLGY | Known *trans*-substrate |
| 2420 | DVVCCSMSYSGY | + | + | + | DVVCC↓SMSYSGY | Known *trans*-substrate |
| 1656 | DLEVVTSTWVGY | - | - | N/A |  | Known *cis*-substrate |
| 946 | GALTGTYVYNGY | - | - | - |  | Negative control |
| 1995 | DFKTWLKAKLGY | - | - | - |  | Negative control |
| 2172a | PDVAVLTSMLGY | + | - | + | PDVAVLT↓SMLGY | Newly identified site |
| 2172b | VAVLTSMLTDGY | + | - | + | VAVLT↓SMLTDGY | Newly identified site |
| 676 | QWQVLPCSFTGY | +/- | - | + | QWQVLPC↓SFTGY | Assay positive in two out of three time points |
| 1429a | DVSVIPTSGDGY | +/- | - | - |  | Assay positive in two out of three time points |
| 1429b | IPTSGDVVVVGY | +/- | - | - |  | Assay positive in two out of three time points |
